# The challenges of big data

**DOI:** 10.1242/dmm.025585

**Published:** 2016-05-01

**Authors:** Elaine R. Mardis

**Affiliations:** McDonnell Genome Institute at Washington University School of Medicine, St Louis, MO 63108, USA

## Abstract

The largely untapped potential of big data analytics is a feeding frenzy that has been fueled by the production of many next-generation-sequencing-based data sets that are seeking to answer long-held questions about the biology of human diseases. Although these approaches are likely to be a powerful means of revealing new biological insights, there are a number of substantial challenges that currently hamper efforts to harness the power of big data. This Editorial outlines several such challenges as a means of illustrating that the path to big data revelations is paved with perils that the scientific community must overcome to pursue this important quest.

## Abstract (precis)

**Summary:** Next-generation-sequencing-based data sets provide a source of ‘big data’ for analytic approaches that could have a powerful impact on biomedical research. In this Editorial, Elaine Mardis highlights the challenges involved in realizing the full potential of big data analytics.

## Introduction

The term ‘big data’, often used in modern-day scientific conversations, publications and in the scientific press, has primarily emerged as a by-product of the widespread use of next-generation sequencing (NGS) to generate large data sets in biomedical research. Growth in the number and magnitude of such data sets in recent years is due to the constant, better-than-Moore's-law improvements in per-instrument data output, the increasingly common use of these instruments and the shrinking costs of data generation. Furthermore, the increasing number of molecular techniques that transition into an experimental data set produced by NGS is astounding, and contributes substantially as a ‘big’ multiplier. These techniques include straightforward assays such as RNA sequencing (RNA-seq) and bisulfite conversion sequencing (methyl-seq), and significantly more complex assays such as three-dimensional chromatin conformation sequencing (3Cseq) and chromatin immunoprecipitation RNA sequencing (ChIRP-seq), among many others. Each new sequencing technique necessitates the accompanying evolution of computational analysis paradigms that interpret the data as a route toward further defining the underlying biology. Now, with the wealth of data in-hand and new data sets being produced at an ever-increasing rate, initiatives such as the National Institutes of Health (NIH)-funded ‘Big Data to Knowledge’ (BD2K) are underway to develop, implement and prove the value of big data in our quest for biological knowledge (https://datascience.nih.gov/bd2k).

Although the current potential for translating results from big-data analysis into biological understanding is unprecedented, there are aspects of big data that might confound our best intentions to gain knowledge from it and therefore must be recognized. These confounders include the ever-changing landscape of next-generation sequencers and their associated characteristics, evolving data analysis paradigms, challenges of computational infrastructure, data sharing and data access, and – crucially – our ability to integrate data sets and their analysis toward an improved understanding of the biology in question.


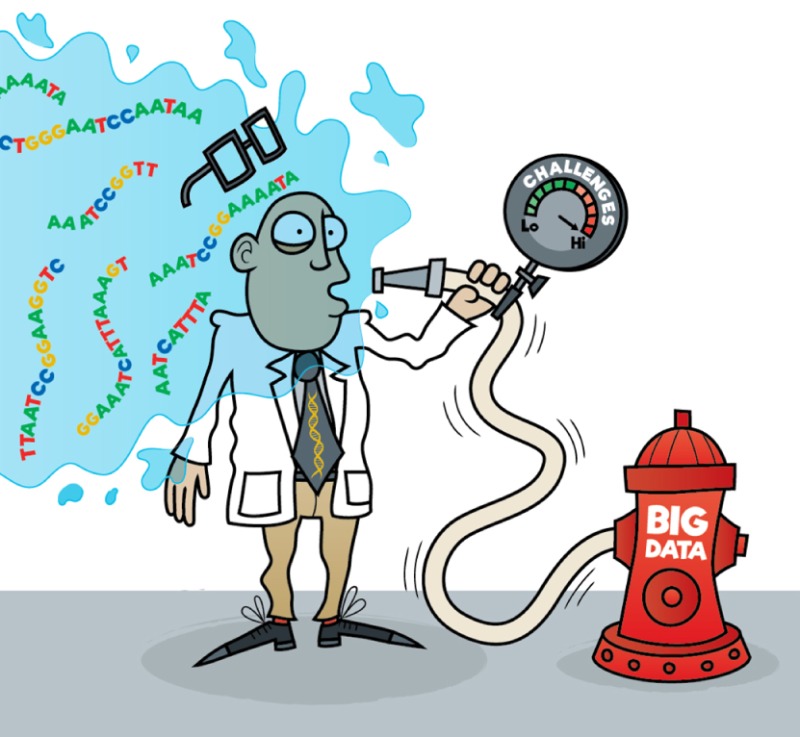


## NGS: the great ‘equalizer’?

The juggernaut that NGS has become began with the introduction of the 454 platform in mid-2005 (Margulies et al., 2005), and underwent quantum leaps in the ensuing 10 years, particularly the sequencing-by-synthesis (SBS) approach first pioneered by Solexa and fully developed by Illumina (Bentley et al., 2008). Ironically, only a decade later, the 454 platform is no longer commercially available, similar to several other NGS instrument platforms that were introduced subsequently (SOLID, Helicos, Polinator). At the same time, single-molecule sequencing platforms have been commercialized and achieved some measure of customer utilization (Pacific Biosciences, Oxford Nanopore). Thus, there has been and will continue to be a variety of chemistries and platforms contributing to big-data generation. This mixed bag of data formats, data qualities, read lengths, single- vs paired-end reads, and other idiosyncrasies limits the cross-comparability of NGS data sets that could contribute to big data analyses. When considered in light of the current-day costs of sequence data generation, the idea of regenerating data with a newer platform rather than analyzing older data begins to make incredibly good sense. As an extreme example, we sequenced the first cancer genome (using tumor cells from an individual with acute myeloid leukemia) and its matched normal counterpart (using cells from the person's skin) with 35 bp single-ended NGS reads at a cost of approximately US$1M (Ley et al., 2008; Mardis, 2014). We achieved ∼30-fold coverage of each genome, using the Solexa approach. At present day, the same tumor and normal genome coverage in paired-end reads (see below) of 150 bp length would cost ∼US$3000. Of course, regeneration of data requires that sufficient tumor and normal samples or isolated DNAs exist for new library construction.

The majority of big data sets have been generated by Illumina SBS technology, which has, since its initial introduction in 2006, developed episodically yet dramatically, by what one might refer to as a ‘punctuated evolution’ model. These developments have entailed improvements in base-calling accuracy, read length and the generation of reads from each end of the library fragment (paired-end reads). Although the trend is undoubtedly in the correct direction of improving data quality, comparability of newer to older data sets is again challenged because the improvements in read quality and length have been dramatic over a relatively short timeframe. In high-scale data generation, characteristics that can negatively impact big data sets include so-called batch effects, which inevitably occur when large numbers of samples are processed through multiple data-generation steps using scaled-up reagent batches.

Another set of obstacles arises owing to the need for short-read data to be aligned to a reference genome prior to variant identification. The completeness and quality of most reference genomes, including the Human Reference Genome, are continually being improved, with new genes being identified and problematic regions being solved to high resolution. Although these improvements are desirable for many reasons, in many analytical paradigms, changes to a reference genome mean that realignment of read data to the latest reference version followed by re-interpretation based on current genome annotation is required. These maneuvers require time and expense to obtain a current set of variants and annotations. Furthermore, algorithms and data analysis pipelines are almost constantly evolving, with efforts to include best-in-class alignment for a given data type, high sensitivity to detect variants of all types, and so on. Ultimately, these factors create huge demands on computational and data-storage infrastructures. Although shifting the burden of re-processing to cloud computing – networks of remote servers hosted on the internet – promises to help address this problem, its adoption by funding agencies and by academic and medical institutions remains slow because of concerns about data privacy protection in the cloud environment.

A related concern is the use of a ‘fixed’ reference human genome that is meant to represent essentially all human genomes. We clearly recognize that this is not representative, yet by aligning reads to a fixed reference sequence rather than by producing a *de novo* assembly of the reads, we accept the fact that any novel content in the sequenced genome will be missed. Ongoing efforts to develop graph-based alignment will bridge this deficit to some extent, as will efforts to improve and diversify the available reference genomes (http://genome.wustl.edu/projects/detail/reference-genomes-improvement/).

## Computing and storage: how much is enough?

As previously alluded to, a commitment to analyze big data requires a commitment to utilize large amounts of computing cycles and storage, regardless of whether the data are generated in-house, imported from external repositories, or both. Downloading data from outside data warehouses requires substantial data-storage resources and significant network bandwidth to complete data transfers in a reasonable timeframe. The computational cycles required to compute on big data also scale with the data volumes as well as the types and complexities of algorithms used in the analyses and their relative efficiencies. Trends to help address data volumes include data compression efforts such as CRAM (http://www.ebi.ac.uk/ena/software/cram-toolkit) (Hsi-Yang Fritz et al., 2011). However, these data-compression approaches might complicate comparative analyses when input data include combinations of uncompressed and never-compressed sources. In extreme cases, there might even be batch effects attributable to specific data-compression formats. Addressing this challenge by reprocessing data to eliminate batch effects resulting from compression or differences in specific analytical pipelines adds to the burgeoning costs associated with computing and storage. Cloud-based computing can provide some relief to the challenges of computational cycles and storage, but, as discussed above, is not without its own challenges for research involving human subjects.

## Data access and sharing: what's mine is yours?

Perhaps the most frequent (and irritating) challenge in big-data efforts is the inaccessibility of data sets from external sources. Let's imagine that you want to download all exome sequencing data from all breast cancers sequenced using NGS, together with available treatment and outcome data (i.e. progression-free survival vs death from disease), to identify whether there are specific genes or mutations or combinations of specific genes/mutations that predict poorer outcome. After researching PubMed to identify relevant published studies, and with study numbers for those with publicly accessible data in-hand, downloading the NGS data and associated clinical metadata from the indicated repositories would be the next seemingly straightforward step. In practice, the road can be full of obstacles.

Sharing data can pose substantial challenges, including the need for inter- and intra-institutional legal documents such as material transfer agreements (MTAs), data use agreements (DUAs), confidentiality and disclosure agreements (CDAs) and other agreements that must meet the approval of and be signed by the appropriate institutional officials. For data derived from human subjects, an in-house Institutional Review Board (IRB) approval for the study typically must be in place, which will require evaluation and approval of the study and corresponding consent documents by the institutional IRB and/or Human Research Protections Office (HRPO). Including the clinical metadata needed to derive prognostic information about each patient in our imaginary big-data-mining study could be complicated by the need to obtain outcomes data from clinical trials that are either not yet completed or published (and hence not released), or necessitate gaining access to electronic health records (EHRs). Owing to the nature of clinical data and concerns about genomic data privacy, EHR access can be exceptionally challenging. New sources of behavioral and health-monitoring data types that will inform big-data analysis will also become important for accession and incorporation into these efforts, including data from wearable devices (e.g. Fitbit^®^ and other activity trackers). Similar concerns about data privacy arise from these sources, and must also be taken into account.

Accessing data from public repositories also poses multiple difficulties, in spite of the best intentions to make data sharing an obligation of those who receive funding from government agencies, most of which mandate data deposition as a requisite for funding. Open-access journals such as Disease Models & Mechanisms (DMM) have also made efforts to improve access to data by requiring its deposition in cases in which an appropriate repository exists. Challenges include the difficulty of uploading data, the rate at which data are actually available (this is confounded both by researchers being slow to transfer data and government-funded repositories being overburdened/slow to post the data to the study location once it is in-house), the poor organization of data repositories that make the data and/or associated metadata difficult-to-impossible to locate, and long waits for approval to access data from restricted access portals (for human subjects data) once it is available. In an attempt to address some of these challenges, DMM recently partnered with Dryad, a general-purpose digital repository, to integrate data deposition with manuscript submission and thus make data sharing more seamless for researchers (http://dmm.biologists.org/content/news#dryad).

## Data integration: the ultimate challenge?

Assuming that all the aforementioned hurdles can be overcome, and with data in-hand to complete our big-data analysis of breast cancer outcomes in the context of prognostic genes and their mutations, how do we integrate big data with clinical data to truly obtain new knowledge or information that can be further tested in the appropriate follow-on study? Although the correlation of mutational status to outcomes is straightforward by Kaplan–Meier analysis, the integration of clinical metadata from multiple studies can be difficult for several reasons, including data-type and format differences between studies, differential nomenclature of similar data fields in the metadata, or missing data fields in some metadata sets. One effort to help address these issues is being led by the Genomic Alliance for Genomes and Health (GA4GH), who are promoting the idea of using restricted vocabularies to label metadata fields and thereby facilitate data integration (http://ga4gh.org/#/metadata-team).

Realistically, however, the desire for integration is not to rely on straightforward one-to-one correlation analyses, but rather to incorporate multiple data types into a single analytical effort with the aim of achieving a more comprehensive knowledge. Returning to our previous example to illustrate this point, perhaps what is needed to fully understand the contribution of genes to outcome in breast cancer goes beyond a simple analysis of mutations. In this example, we would want to incorporate additional data types that characterize other modifications to genes – such as amplification or deletion events, silencing or overexpression of genes by altered methylation or chromatin accessibility, mutations in transcription-factor-binding sites or other regulatory sequences that result in altered expression levels of the gene, and so on. This type of multiplex analysis is more than an unresolved problem; it is a critical step toward turning big data into knowledge. That said, the challenge of big data is out there. The many hurdles that confound big data analytics are not insurmountable; rather, they represent obstacles that can and likely will be sorted out in the same way that other large-scale problems have been overcome – by simply presenting themselves as challenges toward which intelligent and innovative scientists will toil away until solved.
